# Ceftobiprole Medocaril Is an Effective Post-Exposure Treatment in the Fischer 344 Rat Model of Pneumonic Tularemia

**DOI:** 10.3390/antibiotics12081337

**Published:** 2023-08-19

**Authors:** Mark M. Hahn, Cheryl A. Triplett, Michael S. Anderson, Jennifer I. Smart, Karine Litherland, Stephen Keech, Franziska von Siebenthal, Mark Jones, Andrew J. Phipps, Lisa N. Henning

**Affiliations:** 1 Battelle, West Jefferson, OH 43162, USA; 2 Basilea Pharmaceutica International Ltd., 4123 Allschwil, Switzerland; 3 Tunnell Government Services, Bethesda, MD 20817, USA

**Keywords:** ceftobiprole medocaril, *Francisella tularensis*, preclinical models, pneumonic tularemia, biodefense and biothreat preparedness

## Abstract

*Francisella tularensis* subspecies *tularensis* is a category-A biothreat agent that can cause lethal tularemia. Ceftobiprole medocaril is being explored as a medical countermeasure for the treatment of pneumonic tularemia. The efficacy of ceftobiprole medocaril against inhalational tularemia was evaluated in the Fischer 344 rat model of infection. The dose was expected to be effective against *F. tularensis* isolates with ceftobiprole minimum inhibitory concentrations ≤0.5 µg/mL. Animals treated with ceftobiprole medocaril exhibited a 92% survival rate 31 days post-challenge, identical to the survival of levofloxacin-treated rats. By comparison, rats receiving placebo experienced 100% mortality. Terminally collected blood, liver, lung, and spleen samples confirmed disseminated *F. tularensis* infections in most animals that died prior to completing treatments (placebo animals and a rat treated with ceftobiprole medocaril), although levels of bacteria detected in the placebo samples were significantly elevated compared to the ceftobiprole-medocaril-treated group geometric mean. Furthermore, no evidence of infection was detected in any rat that completed ceftobiprole medocaril or levofloxacin treatment and survived to the end of the post-treatment observation period. Overall, survival rates, body weights, and bacterial burdens consistently demonstrated that treatment with ceftobiprole medocaril is efficacious against otherwise fatal cases of pneumonic tularemia in the rat model.

## 1. Introduction

*Francisella tularensis*, the causative agent of tularemia, is a non-motile gram-negative facultative intracellular coccobacillus. The *F. tularensis* subspecies *tularensis* Schu S4 strain is one of the most infectious pathogenic bacteria known, as the inhalation of ≤10 organisms can cause disease with a high mortality rate [1]. *F. tularensis* is categorized as a priority category-A threat agent by the Centers for Disease Control and Prevention (CDC) because it has many characteristics that make it a potential agent to be used as a biological weapon, such as high infectivity, ease of growth in large quantities, and viability in the environment or in weaponized preparations. Inhalation of *F. tularensis* can cause lethal cases of pneumonia, shock, and respiratory failure, making aerosol dispersion a likely method for a biological attack. CDC models to estimate the impact of an intentional release of aerosolized *F. tularensis* over a city of 1.2 million people predict exposure of at least 200,000 people, of whom 4800 (2.3%) would become ill and 1700 (35.4% of ill patients) would die without appropriate antibiotic treatment [2].

Although doxycycline is the only FDA-approved antibiotic for the treatment of tularemia, ciprofloxacin and gentamicin have also been used successfully [3,4,5,6]. However, cure rates vary from 60 to 100% depending on the antibiotic used, the time to treatment, duration of therapy, and patient complications such as lymph node suppuration [5,7]. Early treatment with appropriate antibiotics is a critical factor in survival, as mortality may be reduced from 30–60% (untreated respiratory exposures) to 1–2.5% [8,9]. To reduce the possible impact of an intentional release, which may involve *F. tularensis* strains with naturally acquired or artificially transferred mechanisms of resistance to first-line antibiotics, developing and maintaining a diverse supply of antibiotics with clinical efficacy against *F. tularensis* is a critical risk mitigation measure [9,10,11,12].

Ceftobiprole medocaril (the prodrug of ceftobiprole) is a potential alternative therapy for the treatment of tularemia because of its clinical profile against pneumonic infections. Ceftobiprole is a broad-spectrum cephalosporin with in vitro and in vivo activity against gram-positive and gram-negative pathogens such as methicillin-resistant *Staphylococcus aureus* and non-extended spectrum β-lactamase-producing *Enterobacterales* [13,14,15,16,17]. Furthermore, ceftobiprole medocaril is indicated for the treatment of hospital-acquired pneumonia (HAP; excluding ventilator-associated pneumonia) [18,19] and community-acquired pneumonia (CAP) [20,21] caused by susceptible strains of *S. aureus* (including MRSA), *Streptococcus pneumoniae*, *Escherichia coli*, and *Klebsiella pneumoniae*, or *Haemophilus influenzae* (CAP only) [22]. Finally, ceftobiprole medocaril has also completed pivotal Phase III studies for the treatment of *S. aureus* bacteremia [23,24] and acute bacterial skin and skin structure infections [25,26].

The low natural incidence of *F. tularensis* infections limits the ability to evaluate the clinical efficacy of potential therapies. Thus, approval of ceftobiprole medocaril to treat tularemia will depend on subpart I of the FDA Animal Rule [27] with initial evaluation occurring in a small animal model. A female Fischer 344 rat model of infection has several advantages for evaluating the efficacy of ceftobiprole medocaril against *F. tularensis*. Rats challenged with *F. tularensis* via the intratracheal route demonstrate susceptibility to infection that is comparable to human cases [28,29] and a natural history study of pneumonic tularemia by Hutt et al. demonstrated that pathogenesis is consistent with human disease [30], suggesting that inhalational challenge of female Fischer 344 rats effectively models pneumonic tularemia in humans. This animal model is considered optimal for the evaluation of ceftobiprole medocaril because preliminary exposure modeling demonstrated that the pharmacokinetic profile of ceftobiprole in female Fischer 344 rats could be adapted to provide ceftobiprole exposures that mimic the human concentration versus time profile achieved by the clinical dosing regimen of 500 mg q8h (infused over 2 h). Therefore, the dosing regimen of 110 mg/kg ceftobiprole (i.e., 145 mg/kg ceftobiprole medocaril) q8h (infused over 1 h) was selected for this study as modeling predicted this dose to mimic the human equivalent exposure with the clinical dosing regimen. Although the ceftobiprole pharmacodynamic target has not been established for *F. tularensis*, this dose is predicted to be effective against *F. tularensis* isolates exhibiting ceftobiprole minimum inhibitory concentrations (MICs) of up to 0.5 µg/mL because the time above this MIC (*f*T > MIC) was estimated to be 61% of the dosing interval. Finally, the half-life (t_1/2_) of ceftobiprole in female Fischer 344 rats (0.8 h) offers an advantage to mouse models, which exhibit the rapid elimination of ceftobiprole (t_1/2_: 0.2–0.3 h, unpublished data); a characteristic that would require a prohibitive number of daily infusions to achieve a humanized dose in mice.

Considering the clinical experience with ceftobiprole medocaril, the pharmacokinetic parameters in female Fischer 344 rats, and the previously reported in vitro activity against *F. tularensis* Schu S4 (ceftobiprole MIC of 0.03–0.06 µg/mL) [31], we hypothesized that post-exposure treatment with ceftobiprole medocaril (1-h infusions of 145 mg/kg, q8h for 14 days) would be an effective measure against inhalational tularemia. To test our hypothesis, we established lethal inhalational exposure doses of *F. tularensis* Schu S4 and evaluated the outcomes of rats treated with ceftobiprole medocaril or levofloxacin (as a positive control treatment) [32]. Our data demonstrate for the first time that treatment with ceftobiprole medocaril prevents mortality, limits severe clinical disease, and facilitates clearance of *F. tularensis* infection in the rat model of infection.

## 2. Materials and Methods

### 2.1. Animals

A total of 52 female Fischer 344 rats (Charles River; Wilmington, MA, USA) were used in this study and each rat had a body weight of 150–200 g on the day of challenge. This study was conducted in two phases. Phase I (model refinement) utilized 20 rats and Phase II (treatment evaluation) used 32 rats. All animal procedures were approved by the Battelle Institutional Animal Care and Use Committee (IACUC). General procedures for animal care and housing met AAALAC International recommendations as described in *The Guide for the Care and Use of Laboratory Animals* [12] at the time of conduct. Food and water were available ad libitum. Animals were quarantined for 7 days and acclimated to the BSL-3 facility and restraint tubes prior to challenges.

### 2.2. Phase I (Model Refinement)

The 20 rats for Phase I were group-housed and assigned to one of two challenge groups (10 rats per group) with a target aerosol exposure of 100 *F. tularensis* CFUs/animal (Phase 1, Group 1) or 1000 *F. tularensis* CFUs/animal (Phase I, Group 2).

#### 2.2.1. Preparation of *F. tularensis* for Animal Challenge

*F. tularensis* subspecies *tularensis* strain Schu S4 was prepared for aerosol challenge from a submaster cell bank acquired from BEI resources (Manassas, VA, USA; catalog number NR-10492). Briefly, colonies were isolated on Glucose Cysteine Blood Agar (Northeast Laboratory Services; Waterville, ME, USA) then transferred to cation-adjusted Mueller–Hinton broth (Remel; San Diego, CA, USA) supplemented with 0.1% glucose and 2% IsoVitaleX (BD Diagnostics; Franklin Lakes, NJ, USA) for overnight broth culture at 37 °C ± 2 °C and shaking set to 200 rpm. The culture was washed twice by centrifugation and the bacterial pellet was resuspended in Brain Heart Infusion Broth (BD Diagnostics; Franklin Lakes, NJ, USA). The final stock suspension was confirmed as gram-negative, evaluated for a positive agglutination reaction against *F. tularensis* antibodies, and then normalized by optical density at 600 nm (OD_600_) and dilution in Brain Heart Infusion Broth to achieve appropriate target dose for aerosol exposure. 

#### 2.2.2. Inhalational Challenge

Rats were challenged with aerosolized *F. tularensis* using a nose-only exposure chamber (CH Technologies, Inc., Westwood, NJ, USA) designed to simultaneously expose multiple rats to a homogenous, small-particle aerosol (defined as 1–5 µm) to facilitate deep lung deposition [30,33]. Aerosols were generated using a 6-jet Collison nebulizer and challenges were performed as previously described [34]. To determine the aerosol particle size distribution, air samples from the environment within the exposure chamber were analyzed using an Aerodynamic Particle Sizer^®^ (APS) Spectrometer (Model 3321, TSI Inc.; Shoreview, MN, USA) with an aerosol diluter (Model 3302A, TSI Inc.; Shoreview, MN, USA). A total of 10 rats were challenged at a time (based on target inhaled exposure of 100 or 1000 CFUs/rat) and all 20 rats were challenged on the same day. Targeted inhaled exposure doses were achieved by controlling the aerosol concentration of *F. tularensis* and the time of exposure (approximately 10 min). Actual inhaled exposure doses were determined for each challenge run using Guyton’s formula [35] to determine the respired minute volume, the group mean body weight of rats in the exposure run, and the actual concentration of *F. tularensis* based on the impinger sample enumeration results.

#### 2.2.3. Model Assessment

All rats were observed twice daily for clinical signs of disease or mortality throughout the study except for days 1–7 post-challenge and on the final day (16 days post-challenge). Starting at 08:00 h, 1 day post-challenge and ending at 16:00 h, 7 days post-challenge, animals were monitored three times daily at 00:00 h, 08:00 h, and 16:00 h. A single observation was performed on the final day prior to scheduled euthanasia/study termination.

Animals determined to be in a moribund state and those surviving to the end of the observation period were humanely anesthetized and euthanized. IACUC-approved early termination criteria included the following signs: any rat that was observed as moribund, unresponsive to stimuli, non-ambulatory, experiencing convulsions, or ≥25% body weight loss (compared to pre-challenge baseline) in combination with severe signs of illness.

In addition to daily monitoring for mortality and clinical signs of disease, animal body weights were collected on day 0 (prior to challenge) and 7 days and 14 days post-challenge.

### 2.3. Phase II (Treatment Evaluation)

Phase II rats were procured with a surgically implanted Vascular Access Button™ (VAB) (Instech; Plymouth Meeting, PA, USA) and single-housed to reduce potential complications at the surgical sites due to interference from other animals. The 32 rats for Phase II were assigned to one of three treatment groups with a target aerosol exposure of 1000 *F. tularensis* CFUs/animal for all three groups. Phase II, Group 1 (treatment group) consisted of 12 rats designated to receive ceftobiprole medocaril infusions. Phase II, Group 2 (positive control group) consisted of 12 rats designated to receive levofloxacin boluses. Phase II, Group 3 (placebo group) consisted of 8 rats designated to receive infusions of vehicle (ceftobiprole medocaril preparation solution; described below). The Phase II study was performed in the BSL-3 laboratory at the Battelle Biomedical Research Center.

#### 2.3.1. Preparation of *F. tularensis* for Animal Challenges and Inhalational Challenges

Phase II experimental procedures were conducted using two equal cohorts (Cohort A or B) consisting of six Group 1 animals, six Group 2 animals, and four Group 3 animals. All 16 rats in a single cohort were exposed simultaneously with a target exposure of 1000 CFUs/animal. Cohorts were challenged on separate days (Challenge Day A [CDA] or Challenge Day B [CDB]). *F. tularensis* challenge inocula was freshly prepared for each challenge day and inhalational exposures were conducted as described for Phase I challenges.

#### 2.3.2. Minimum Inhibitory Concentration Testing by Broth Macrodilution

The MIC of ceftobiprole against the *F. tularensis* Schu S4 isolates used to prepare challenge inocula for each Phase II challenge day was evaluated by broth macrodilution using a method derived from the Clinical and Laboratory Standards Institute (CLSI) broth macrodilution methods [31]. *S. aureus* ATCC 29213 and ciprofloxacin were included in each test as a quality control organism or antibiotic (respectively), allowing for comparison of results to CLSI reference data. All growth for MIC testing was conducted in Modified Cysteine Partial Hydrolysate (MCPH) broth free of cysteine (MCPH^−^). Broth was prepared in deionized water with the following nutrient concentrations: 6.25 g/L Difco Bacto yeast extract (Gibco; Carlsbad, CA, USA), 12.5 g/L casein hydrolysate (Gibco; Carlsbad, CA, USA), 6.25 g/L sodium chloride, 8 mmol K_2_HPO_4_, 24.5 mmol KH_2_PO_4_, and 0.00025% thiamine hydrochloride (*w*/*v*). pH was adjusted to ~6.7 prior to autoclave sterilization.

A 10 mg/mL ciprofloxacin (United States Pharmacopeia [USP]; Frederick, MD, USA; catalog number 1134313) stock solution was prepared in deionized water supplemented with 0.1 M sodium hydroxide and either used fresh for the MIC assay or frozen at −85 °C to −60 °C for use at a later date. A 10 mg/mL ceftobiprole (active moiety of ceftobiprole medocaril) stock solution was prepared fresh on the day of each assay by reconstituting 10 mg ceftobiprole (Compound ID BAL0009141-00, Lot number 08004R25F) in a solution of 990 µL dimethyl sulfoxide (DMSO) plus 10 µL acetic acid and vortexing for 15 min.

Fresh isolates from each challenge day were prepared on Chocolate II agar supplemented with hemoglobin and 2% IsoVitaleX (BD Diagnostics; Franklin Lakes, NJ, USA) and colonies of *S. aureus* were isolated on Tryptic Soy Agar (Hardy Diagnostics; Santa Maria, CA, USA). An isolated colony of *S. aureus* was then used to inoculate 5 mL MCPH^−^ and incubated at 37 °C ± 2 °C with shaking agitation (200 rpm) for 16–24 h.

On the day of each assay, working stock solutions of ceftobiprole (256 µg/mL) and ciprofloxacin (32 µg/mL) were prepared in MCPH^−^. Macrobroth dilution tubes were filled with 5 mL MCPH^−^ to prepare 2-fold dilution series of ceftobiprole or ciprofloxacin.

To generate a stock inoculum of *F. tularensis*, isolated colonies were suspended in MCPH^−^ and the suspension was normalized to an optical density at 600 nm (OD_600_) of 0.100 ± 0.05 and then serially diluted 1:1000 in MCPH^−^. The *S. aureus* stock inoculum was produced by transferring the overnight broth culture to MCPH^−^ at a ratio of 30 µL overnight broth culture to 15 mL MCPH^−^. 5 mL of the *F. tularensis* or *S. aureus* stock inoculum was then added to all 12 tubes of a ceftobiprole dilution series (final range 64—0.03 µg/mL) or a ciprofloxacin dilution series (final range 8—0.004 µg/mL) (the final target concentration of *F. tularensis* was 4.0 × 10^5^ CFUs/mL). Positive growth controls were also prepared by culturing each bacterial stock in antibiotic-free conditions. The following negative controls were also included with each assay to verify sterility of all reagents and media used: MCPH^−^, MCPH^−^ (64 µg/mL ceftobiprole), and MCPH^−^ (8 µg/mL ciprofloxacin). All macrobroth tubes were incubated at 37 °C ± 2 °C with shaking agitation (200 rpm). MIC of each antibiotic was determined by visual inspection for growth in each macrobroth tube at 24 h ± 2 h and 48 h ± 2 h. Observations of growth and inhibition were made by comparison to the positive growth controls.

#### 2.3.3. Antibiotics and Formulation Preparation for Animal Experiments

All antibiotic and formulation reagents were pharmaceutical grade. Vehicle was prepared by supplementing 5% Dextrose Injection, USP (ICU Medical; Austin, TX, USA; lot number 5751685) with 5% (*v*/*v*) DMSO, USP (Medisca; Montreal, QC, Canada; lot number 184203) and stored at room temperature. Ceftobiprole medocaril, 500 mg vials (Nipro Pharma Corporation; Osaka, Japan; lot number 20P03) (containing 706 mg of ceftobiprole medocaril sodium) were stored at 2–8 °C until reconstitution and dilution with vehicle. Briefly, stock vials were reconstituted using a sterile needle and syringe to pierce the rubber stopper and inject 20 mL of vehicle. Contents were shaken vigorously until complete dissolution was observed (up to 10 min), then placed at rest to allow foam to dissipate. Solutions were visually inspected to ensure the absence of particulate matter, then stock solutions were further diluted in vehicle to 15 mg/mL and mixed by gently inverting 5–10 times. Dilute doses were stored at 2–8 °C, protected from light, and were used for infusions only if the infusion would be complete within 24 h of dilution to 15 mg/mL based on stability recommendations from the manufacturer. Levofloxacin Injection (5 mg/mL) (Hikma Pharmaceuticals; London, UK; lot number 2107105.1) was stored at room temperature, protected from light, and no additional preparation steps were conducted prior to administration.

#### 2.3.4. Antibiotic Administration

Ceftobiprole medocaril and placebo treatments were administered by 60 min (±10 min) intravenous (IV) infusions three times daily for 14 consecutive days starting 3 days post-challenge. A total of 42 doses were administered to rats surviving to the end of the 14-day treatment period. Infusions were administered at approximately 8 h intervals, based on the start time of the previous treatment. Ceftobiprole medocaril doses were administered at 145 mg/kg (110 mg/kg of active moiety) based on day 0 (pre-challenge) body weight and placebo doses were administered at 9.67 mL/kg (equivalent volume/kg as ceftobiprole medocaril infusions) based on day 0 body weight. Levofloxacin was administered by slow IV bolus (delivered over 3–5 min) twice daily for 14 consecutive days starting 3 days post-challenge. A total of 28 doses were administered to rats surviving to the end of the treatment period. Each bolus was administered at approximately 12 h intervals, based on the start time of the previous treatment. Levofloxacin doses were administered at 50 mg/kg based on day 0 body weight. All infusion and bolus doses were calculated using individual animal body weight and remained constant for all treatments, regardless of any changes in body weight.

Infusions of ceftobiprole medocaril and vehicle were administered via a BS-8000 Syringe Pump (Braintree Scientific; Braintree, MA, USA) connected to the implanted VAB via a sterile polyurethane line protected by a Snap-in Spring Swivel Mount tether (Instech) designed to allow for free movement of the animal in its cage. Levofloxacin bolus injections were manually administered using a syringe and PinPort adapter (Instech) connected directly to the VAB.

#### 2.3.5. Clinical Observations and Body Weights

All rats were observed twice daily for clinical signs of disease or mortality throughout the study except for days 1–7 post-challenge and on the final day (31 days post-challenge). Starting at 08:00 h, 1 day post-challenge and ending at 16:00 h, 7 days post-challenge, animals were monitored three times daily at 00:00 h, 08:00 h, and 16:00 h. A single observation was performed on the final day prior to scheduled euthanasia/study termination. The scheduled termination on day 31 was to allow for a 14-day observation period following the completion of all planned antibiotic or placebo treatments to potentially detect any latent infections.

Animals determined to be in a moribund state and those surviving to the end of the observation period were humanely anesthetized and euthanized as described for Phase I.

Animal body weights were collected daily on days 0–17 (day 0 weight collected prior to challenge) to correspond with the post-exposure treatment period (days 3–17 post-challenge), then weekly thereafter (days 24 and 31 post-challenge). Additional weight measurements were made as needed for veterinary monitoring purposes on rats that appeared to be losing weight over the course of the study.

#### 2.3.6. Bacterial Enumeration

Upon death (either during the course of the study or at scheduled termination 31 days post-challenge), blood and tissue samples were collected from each animal to assess bacterial burdens. Briefly, blood was collected for quantitative assessment of bacteremia. *F. tularensis* was enumerated from whole blood samples by triplicate serial dilution and plating on Chocolate II agar supplemented with hemoglobin and 2% IsoVitaleX (BD Diagnostics; Franklin Lakes, NJ, USA). Bacterial burdens in the lung, liver, and spleen were determined by aseptic collection and homogenization of approximately 1 cm^3^ samples followed by triplicate serial dilution and plating on chocolate agar.

### 2.4. Statistical Analysis

All statistical analyses were conducted using SAS^®^ (version 9.4) and R (version 4.1.2) on the 64-bit platform. All results are reported at the 0.05 level of significance.

#### 2.4.1. Survival

The survival rate and corresponding 95% binomial confidence interval were calculated for each group (Phase I and Phase II). In Phase I, a two-sided Boschloo’s test was used to compare survival between the two challenge groups. In Phase II, a one-sided Boschloo’s test was used to compare survival rates between each of the two groups receiving an antibiotic and the placebo group, and a two-sided Boschloo’s test was used to compare survival rates between the two groups receiving an antibiotic. The Bonferroni–Holm multiple comparison procedure was used to control the overall Type I error rate of the three tests at 5%.

The time from challenge until death was calculated for each animal based on challenge time and time of death. Time of death was the time recorded when a moribund animal was euthanized, or when an animal was found dead. Time-to-death analysis compared the survival distributions of challenge groups (Phase I) or treatment groups (Phase II). The median time to death along with a 95% confidence interval was calculated for each group. The log rank test was used to compare times to death between each challenge group or between each of the two groups receiving an antibiotic and the placebo group as well as between the two groups receiving an antibiotic. The Bonferroni–Holm multiple comparison procedure was used to control the overall Type I error rate of the three tests at 5%. Kaplan–Meier curves were plotted by group, with time to death for surviving animals right-censored at the end of the study.

#### 2.4.2. Body Weight

An ANOVA model was fitted to the body weight data with effects for group, study day, and the interaction between group and study day to assess the model assumption of normality and identify potential outliers. Results from the Shapiro–Wilks test and an examination of the normal probability plot were used to assess the model assumption of normality. Deleted studentized residuals, which are the standardized residuals from the model fitted to all data except the current observation, were calculated for each observation. If the absolute value of the deleted studentized residual was greater than four, then the observation was considered a potential outlier. If any potential outliers were identified, then the statistical analysis was performed both with and without these observations to evaluate their effect on the results.

The following ANOVA model was then fitted to the pre-challenge baseline data (day 0, prior to challenge) to determine if there were significant differences among the groups:

Y*_ij_* = μ + Group*_i_* + ε*_ij_* (1)

where Y*_ij_* is the observed body weight result for the *j*th animal in Group *i* (*i* = 1 to 3) at baseline, μ is an overall constant, Group*_i_* is the effect of Group *i*, and ε*_ij_* is the random error left unexplained by the model.

Descriptive statistics, including means and 95% confidence intervals, were calculated by group and study day (Phase I and Phase II). To determine if, for each Phase II group, there was a significant shift from baseline at each study day, the following ANOVA model was fitted:

Y*_dij_* − Y*_bij_* = μ + Group*_i_* + ε*_ij_* (2)

where Y*_dij_* is the observed body weight for the *j*th animal in Group *i* (*i* = 1 to 3) on study day *d*, Y*_bij_* is the observed body weight for the *j*th animal in Group *i* at baseline, μ is an overall constant, Group*_i_* is the effect of Group *i*, and ε*_ij_* is the random error left unexplained by the model.

Least square mean estimates from these models were calculated and approximate t-tests were performed to determine if, for each group, there was a significant shift from baseline at each study day. This tests if the difference between the means at baseline and each study day was significantly different from zero. In addition, pairwise tests were performed to determine which groups had significantly different mean shifts from baseline. Specifically, least square mean differences were estimated, and t-tests were performed to determine if there were significant differences between the groups. The Type I error rate was controlled at no more than 5% using Tukey’s adjustment procedure.

#### 2.4.3. Bacteremia and Bacterial Load

For quantitative assessment of bacteremia in whole blood and tissue burden in the liver, lung, and spleen, geometric means and 95 percent confidence intervals were calculated for each group. For bacteremia, positive samples with a mean colony count less than 25, or a majority of colony counts for a given dilution set less than 25, were analyzed as 250 CFUs/mL based on the limit of quantification (LOQ).

To determine if there were statistically significant differences in mean values between each group receiving an antibiotic and the placebo group as well as between groups receiving an antibiotic, the following analysis of variance (ANOVA) model was fitted for each sample type:

Log_10_(Y*_ij_*) = μ + Group*_i_* + ε*_ij_* (3)

where Log_10_(Y*_ij_*) is the base-10 log-transformed tissue burden or bacteremia result for the *j*th animal in Group *i* (*i* = 1 to 3), μ is an overall constant, Group*_i_* is the effect of Group *i*, and ε*_ij_* is the random error left unexplained by the model. Least square mean differences were estimated from this model, and t-tests were performed to determine if there were significant differences between the groups. The Type I error rate was controlled at no more than 5% for each sample type using Tukey’s adjustment procedure.

## 3. Results

### 3.1. Phase I

#### 3.1.1. Inhalational Challenge

Phase I animals were challenged with a low or high exposure dose of aerosolized *F. tularensis*. Although the actual exposure doses were greater than the targeted exposure doses (likely due to greater-than-targeted concentrations of *F. tularensis* in the nebulizer samples), the actual exposure doses had a difference of approximately one order of magnitude (Table 1). The mass median aerodynamic diameter (MMAD) of particles analyzed during each challenge ranged from 1.22 (Group 2) to 1.24 µm (Group 1) with geometric standard deviation (GSD) of 1.46 or 1.47, respectively. The range of particle sizes detected was <5 µm.

#### 3.1.2. Mortality, Clinical Observations, and Body Weights

**Figure 1:**
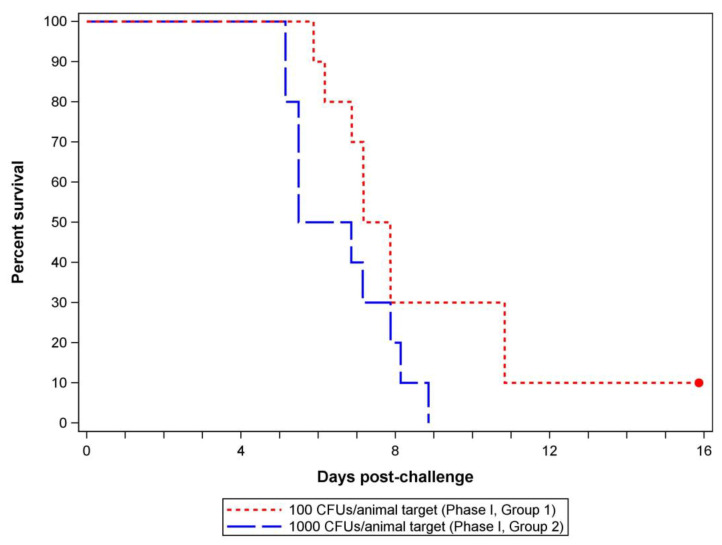
Survival of rats challenged with a low or high exposure dose of *F. tularensis*. Kaplan–Meier curves were generated based on the challenge time and time to death of individual animals (*n* = 10 rats per challenge group). Group 1 survival rate was 10% (95% confidence interval: 0.00, 0.45). Group 2 survival rate was 0% (95% confidence interval: 0.00, 0.31). The red dot indicates scheduled euthanasia/study termination.

A total of 90% of rats (9/10) challenged with the low dose (Group 1, 377 *F. tularensis* CFUs/animal) died during the observation period while one animal (10%) survived to scheduled termination (16 days post-challenge). The median time to death was 180.47 h (95% confidence interval: 141.03, 259.95). Similarly, 100% of the rats (10/10) challenged with the high dose (Group 2, 3515 F. tularensis CFUs/animal) died during the observation period and the median time to death was 148.13 h (95% confidence interval: 123.58, 188.92) (Figure 1). No statistical differences between challenge groups were observed in terms of survival (*p* = 1.0000) or median time to death (*p* = 0.0819). Prior to challenge, animal observations were unremarkable. Challenged animals that succumbed demonstrated a progression of signs of illness (such as rough coat, hunched posture, lethargy, breathing abnormalities [e.g., labored breathing or rapid/increased breathing], and eye discharge) until they were found dead. Abnormal observations were initially reported in the low exposure dose group 5 days post-challenge and in the high exposure dose group 4 days post-challenge.

The body weight of each rat was within 150–200 g at baseline (prior to challenge). Rats lost weight within the first week post-challenge in both the high and low exposure dose groups (Table S1).

### 3.2. Phase II

Based on the statistically equivalent median times to death and mortality rates observed in Phase I testing, a single challenge exposure dose target (1000 *F. tularensis* CFUs/animal) was selected for Phase II testing designed to evaluate the efficacy of post-exposure treatment with ceftobiprole medocaril, levofloxacin, or placebo. A post-treatment monitoring period of 14 days was included in this phase to ensure latent infections not fully cleared by either drug could be detected.

#### 3.2.1. Inhalational Challenge

For Phase II testing, animals were challenged on CDA or CDB (2 cohorts), with each cohort having equal numbers of animals from each treatment group. The inhaled challenge exposures ranged from 1105 CFUs/animal (CDB) to 1244 CFUs/animal (CDA) and the overall average challenge exposure dose for both challenge days was 1174 CFUs/animal.

The MMAD of aerosolized *F. tularensis* ranged from 1.92 on CDB to 2.00 on CDA (geometric standard deviations of 1.41 and 1.37, respectively) and the range of particle sizes detected was generally <5 µm.

#### 3.2.2. Minimum Inhibitory Concentration of Ceftobiprole against *F. tularensis* Used for Inhalational Challenges

MIC testing was conducted for CDA and CDB material in separate assays. Both trials exhibited passing controls (growth in positive growth tubes and no evidence of contamination in negative control tubes). Despite higher-than-expected concentrations of *F. tularensis* or *S. aureus* in each assay (Table 2), the MICs for each quality control condition (ceftobiprole tested against *S. aureus* or ciprofloxacin tested against either species) were on target with previous analysis utilizing the MCPH^−^ broth macrodilution method [31] and results from each day were within one doubling dilution, demonstrating consistency between assays (Table 2). Therefore, the MIC of ceftobiprole against *F. tularensis* Schu S4 was <0.03 µg/mL (Table 2).

#### 3.2.3. Mortality and Clinical Observations

**Figure 2:**
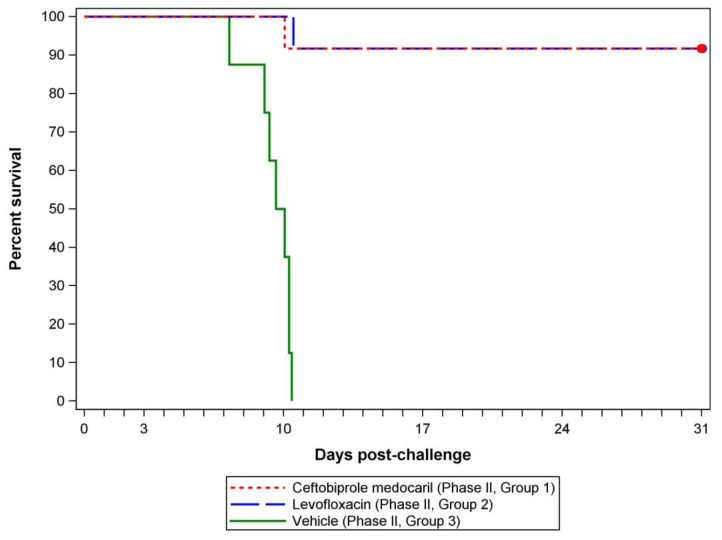
Survival of rats receiving antibiotic or placebo treatments. Kaplan–Meier curves were generated based on the challenge time and time to death of individual animals (*n* = 12, Phase II, Group 1 and Phase II, Group 2; *n* = 8, Phase II, Group 3). Group 1 survival rate was 92% (95% confidence interval: 0.62, 1.00). Group 2 survival rate was 92% (95% confidence interval: 0.62, 1.00). Group 3 survival rate was 0% (95% confidence interval: 0.00, 0.37). The red dot indicates scheduled euthanasia/study termination.

Both the ceftobiprole medocaril treatment group and the levofloxacin control group demonstrated a 92% survival rate (11/12 rats) through the treatment and observation periods (Figure 2). No statistical differences between the ceftobiprole-treated and the levofloxacin-treated groups were observed in terms of overall mortality (*p* = 1.0000) or median time to death (*p* = 0.9755). Median times to death and 95% confidence bounds could not be estimated for the treated groups due to a large portion of those animals surviving to the end of the study. In stark contrast, the placebo group mortality rate was 100% (8/8 rats) and the median time to death was 236.18 h (95% confidence interval: 174.72, 246.72), representing statistically significant differences in survival and time to death compared to either treatment group (*p* < 0.0001, all comparisons) (Figure 2).

Other than scabbing at the VAB surgical site, all rats were observed as normal prior to challenges. Moderate signs of disease, such as eye and nose discharge or eye closures, began to present in animals receiving ceftobiprole medocaril or placebo 4–6 days post-challenge. During the same time frame, a portion of ceftobiprole medocaril treated rats remained normal although the majority had developed rough coats by 6 days post-challenge and rapid breathing was reported for one animal on a single occasion. Beginning 4 days post-challenge (continuing through death), animals receiving placebo infusions experienced more severe signs of disease (in addition to eye and nose related signs), such as a hunched posture, rough coat, lethargy, labored breathing, rapid breathing, and/or reduced feces. No individual receiving placebo infusions presented as normal after 5 days. In the ceftobiprole medocaril treatment group, nose and eye signs resolved by 6 days post-challenge with signs of illness observed from days 7–14 post-challenge generally limited to a rough coat (brief durations of rapid breathing and/or a hunched postures were observed in a few individuals while other animals presented normally).

Animals treated with levofloxacin tended to present normally throughout the course of the study. Signs of disease were limited to one rat that developed a rough coat for two days in the post-treatment observation period (19–20 days post-challenge). Although rats that received ceftobiprole medocaril presented with more signs from days 7–14 post-challenge, similarities to rats that received levofloxacin were observed from days 15–31 post-challenge with all surviving Phase II, Group 1 rats presenting as normal except for a single rat that also developed a rough coat 19–20 days post-challenge.

#### 3.2.4. Body Weights

**Figure 3:**
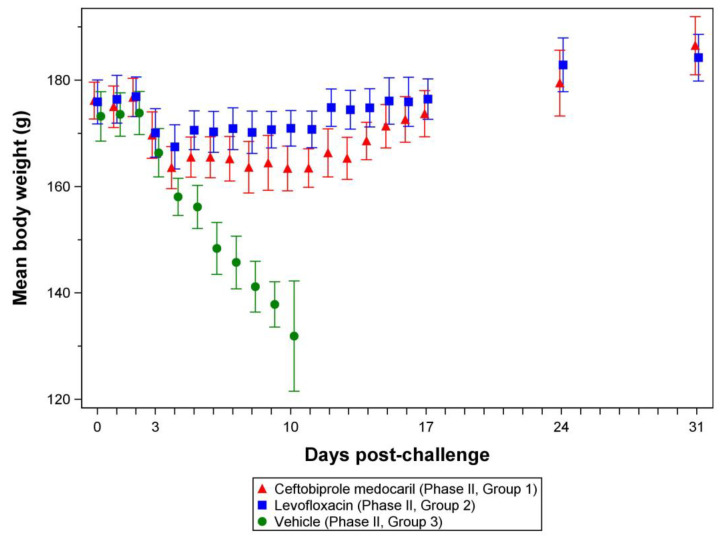
Mean body weight by treatment group. Data represent arithmetic mean and 95% confidence intervals. Results from each group are offset horizontally on each day to differentiate between groups. The actual weight ranges (all groups) were 165.5–185.2 g for challenge day A animals and 167.5–184.7 for challenge day B animals. The model assumption of normality at baseline was reasonable based on the fitted ANOVA models, so no transformations of body weight data were performed for statistical analysis.

All rats had a body weight within 150–200 g at baseline (prior to challenge). No outliers were detected and, based on the ANOVA model fitted, there were no significant differences in mean body weights between any pair of groups. Mean body weights (Figure 3, Table S2) in all groups began to trend downward beginning 3 days post-challenge (Figure 3, Table S3) with significant differences from baseline or between groups emerging 3 or 4 days post-challenge, respectively (Table S3). Rats receiving placebo continued to experience daily weight loss 5–10 days post-challenged with death occurring from days 7–10 post-challenge and a daily mean shift from baseline significantly different from that of the ceftobiprole medocaril or levofloxacin treated groups (*p* value range of <0.0001 to 0.0028) (Table S3). Although the weight loss of animals receiving ceftobiprole medocaril or levofloxacin was not as severe as animals receiving placebo (Figure 3), the magnitude of the ceftobiprole medocaril treatment group mean shifts from baseline were significantly greater than the levofloxacin control group mean shifts from baseline 4–15 days post-challenge (Table S3). However, these disparities dissipated during the late stages of treatment and post-treatment observation period as both groups regained weight with no significant differences between mean shift from baseline 16, 17, and 24 days post-challenge. Furthermore, by 31 days post-challenge, both groups had gained weight (significantly greater than baseline) and, interestingly, the ceftobiprole medocaril treated group mean shift from baseline was significantly greater than the levofloxacin group mean shift from baseline (*p* = 0.0449) (Table S3).

#### 3.2.5. Bacteremia and Tissue Burden

**Figure 4:**
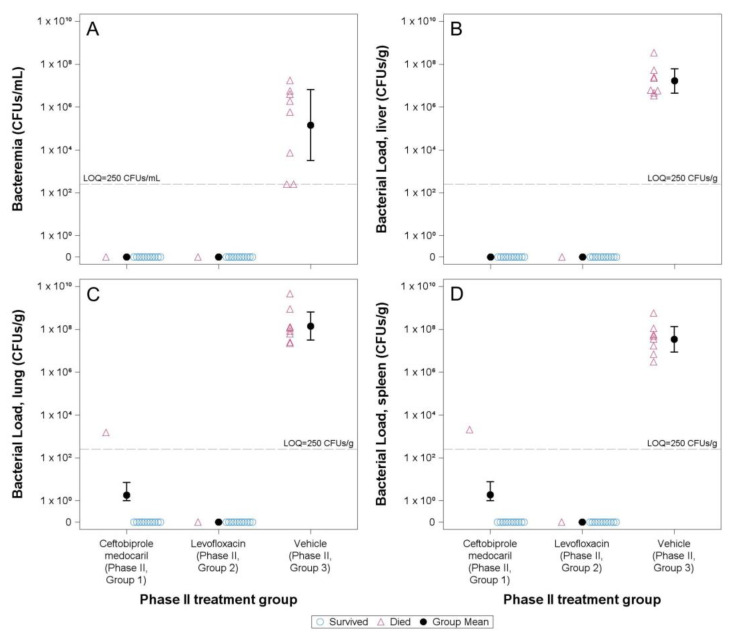
Quantitative bacteremia and tissue burdens by treatment group. Open symbols represent individual animal sample values and solid symbols circles represent group geometric mean values with error bars to indicate the 95% confidence interval. Blue circles indicate the animal survived to the end of the study and magenta triangles indicate the animal died prior to the end of the study. (**A**) Quantitative bacteremia: the concentration of bacteria in individual samples from rats treated with vehicle ranged from below the limit of quantification (<250 CFUs/mL, dashed gray line) to 1.75 × 10^7^ CFUs/mL. (**B**) Liver tissue burden: For the ceftobiprole medocaril treated animal that died prior to the end of the study, the liver sample was positive for *F. tularensis* but below the limit of quantification (<250 CFUs/g, dashed gray line). This sample was excluded from statistical analysis because the normalized tissue burden could not be determined. (**C**) Lung tissue burden (limit of quantification <250 CFUs/g, dashed gray line). (**D**) Splenic tissue burden (limit of quantification <250 CFUs/g, dashed gray line).

Quantitative assessment of bacteremia from blood collected from rats surviving to the end of the observation period or when a rat was found dead did not identify viable bacteria in samples from rats that received ceftobiprole medocaril or levofloxacin treatments (Figure 4A). However, *F. tularensis* was detected in all blood samples from animals treated with vehicle, with a geometric mean concentration of 1.46 × 10^5^ CFUs/mL (95% confidence interval: 3.21 × 10^3^, 6.64 × 10^6^), representing a significant difference from either treatment group (*p* < 0.0001, both comparisons).

*F. tularensis* infection in the liver, lung, and spleen was confirmed in all animals that received placebo infusions (Figure 4B–D), which had geometric mean burdens (and 95% confidence intervals) of 1.67 × 10^7^ (4.55 × 10^6^, 6.13 × 10^7^), 1.42 × 10^8^ (3.15 × 10^7^, 6.45 × 10^8^), or 3.47 × 10^7^ (8.84 × 10^6^, 1.36 × 10^8^) CFUs/g (respectively). For each tissue type, the geometric mean bacterial load was significantly greater in animals receiving placebo compared to either treatment group (*p* < 0.0001, all comparisons) and there were no significant differences between CFU counts in the ceftobiprole medocaril group or the levofloxacin group liver (*p* = 1.0000), lung (*p* = 0.6138), or spleen (*p* = 0.5897) samples. No CFUs were recovered from any rat treated with levofloxacin regardless of whether the rat survived to the end of the study or completed all levofloxacin treatments. By comparison, no CFUs were recovered from rats treated with ceftobiprole medocaril surviving to the end of the study, although *F. tularensis* was detected in each tissue sample collected from the rat (*n* = 1) that was terminated due to observation of convulsions prior to completing all ceftobiprole medocaril infusions. As noted, this difference did not result in statistical significance between the treated groups.

## 4. Discussion

The ability to evaluate the efficacy of ceftobiprole medocaril against *F. tularensis* depends on the use of an appropriate animal model of inhalational exposure. In this multiphase study, Phase I (model refinement) evaluated time to death following two target inhalation exposure doses to determine optimal challenge conditions for Phase II, which evaluated treatment efficacy. Although the actual exposure doses (377 CFUs/animal or 3515 CFUs/animal) were elevated compared to their respective targets (100 CFUs/animal or 1000 CFUs/animal), the relative difference between groups (approximately 10-fold) was consistent with the target difference (Table 1) and, thus, sufficient to make comparisons between exposure dose groups. Furthermore, the level of variation between actual and target exposure doses was similar to the exposure dose variance previously reported by Hutt et al. (more than 3-fold) without impact on animal outcomes [30]. While the exposure dose of *F. tularensis* delivered to the lungs is a critical factor, the characteristics of the aerosols is also an important component [33]. The MMAD and particle sizes detected during each challenge were consistently in the 1–5 µm range, suggesting that particles containing *F. tularensis* were capable of reaching the alveolar region of the lower respiratory tract [33] and providing further support of model appropriateness.

Observations through 16 days post-challenge in Phase I were adequate for documenting the natural course of disease following each exposure dose with 95% of animals succumbing to disease by the end of the period (Figure 1). The higher target exposure dose (1000 *F. tularensis* CFUs/animal) was selected to evaluate the efficacy of ceftobiprole medocaril in Phase II because body weight loss and clinical observations demonstrated steady cachexia until death, with the group receiving a larger exposure dose exhibiting clinical signs and body weight loss at an accelerated rate compared to the lower exposure dose group (Supplemental Table S1). Overall, the time to death range observed in Phase I testing was consistent with prior reports that also used a challenge target of 1000 CFUs/animal (actual exposure dose range of 853–3153 CFUs/rat) [30], providing confidence in model appropriateness for evaluating the efficacy of potential medical countermeasures against inhalational tularemia.

In the Phase II experiment to evaluate the efficacy of post-exposure treatment, the exposure dose for each challenge day and the average exposure dose were above the target exposure dose (1000 CFUs/animal) but within the same inhaled exposure dose range demonstrated in Phase I testing (300–3000 CFUs/animal) to cause 90–100% mortality in untreated rats by 11 days post-challenge (Table 1, Figure 1). Additionally, the MMAD and particle size ranges detected on each challenge day were consistent between days and with Phase I challenges, demonstrating once again that *F. tularensis*-containing particles were likely deposited in the alveolar region of the lower respiratory tract [33]. Consistently, all rats (100%) that received placebo experienced mortality by 10 days post-challenge (Figure 2), indicating that lethal doses were delivered during Phase II challenges. Although the time to death of these animals was slightly delayed compared to Phase I, Group 2 animals, this shift was minor and could have been a result of the exposure dose variations and/or due to a modest effect of receiving supplemental hydration from placebo infusions.

Prior to conducting these experiments, PK modeling suggested that ceftobiprole would achieve sufficient concentrations in Fischer 344 rats to treat isolates of *F. tularensis* Schu S4 with a ceftobiprole MIC of up to 0.5 µg/mL. Analysis of the ceftobiprole MIC against the isolates used to prepare challenge inocula used on either challenge day demonstrated MICs below the tested range (<0.03 µg/mL, Table 2). This result is consistent with the observed efficacy of the ceftobiprole treatment group (Figure 2 and Figure 4) and a previous report that developed the method [31] despite the elevated starting inocula concentrations, which have potential to artificially increase reported MICs [36]. Although an exact MIC value was not identified by the tested range, precise MICs could be identified by adjusting the dilution range used for testing.

Survival by treatment group was the main assessment used to determine the efficacy of ceftobiprole medocaril compared to the placebo or positive control groups. All rats receiving placebo were found dead or terminated due to protocol criteria within 10.4 days post-challenge, while animals that received an antibiotic (ceftobiprole medocaril or levofloxacin) experienced nearly identical outcomes with no statistical differences in the survival rate (92%) and a single fatality in each group occurring with a difference of only 0.44 days post-challenge (Figure 2). A distinct difference in the placebo group outcomes was also evident in clinical observations, which recorded severe and persistent signs of illness compared to the treatment and positive control groups. Finally, the 14-day post-treatment observation period (completed by the treatment and positive control group animals only) was sufficiently longer than the 3–5-day incubation period associated with *F. tularensis* clinical manifestations in humans [37] and the 5–9-days to death observed in Phase I of this study, demonstrating no evidence of latent infections.

The absence of significant body weight differences between groups at baseline or prior to beginning treatments (initiated 3 days post-challenge) implies that the initial group mean changes from baseline were associated with the effects of challenge and not with inherent differences among the groups prior to challenge or treatment. Although the ceftobiprole medocaril and levofloxacin treatment groups experienced significant body weight loss during most (levofloxacin group) or all (ceftobiprole medocaril group) of the treatment phase (3–17 days post-challenge), the effects were mild with neither group experiencing changes as severe as animals in the placebo group (Supplemental Table S3). Consistent with overall survival, these trends demonstrate both ceftobiprole and levofloxacin mitigate the most severe forms of pneumonic tularemia.

Analysis of bacterial burdens in blood and tissues demonstrated complete clearance of *F. tularensis* at the site of infection (lungs) as well as systemic (blood and liver) and immunologic (spleen) sites in animals that survived through the observation period (Figure 4). By comparison, animals that received placebo infusions had evidence of severe infection with significantly greater bacterial burdens detected in all blood and tissue samples, confirming mortality in these rats was due to *F. tularensis* infection. Of the two additional animals that died prior to completing treatment (one animal in each treatment group), the enumeration data support differing conclusions. Blood cultures from each animal were negative, indicating neither were experiencing acute septicemia at the time of death. Low levels of infection detected in the liver, lung, and spleen of the rat receiving ceftobiprole medocaril (Figure 4B–D) suggest that this animal had experienced systemic spread from the site of infection, although treatment with ceftobiprole medocaril was supporting the clearance of *F. tularensis* as bacterial burdens were significantly less than those of placebo-treated animals. By comparison, no evidence of infection was detected in any sample collected from the levofloxacin-treated animal (Figure 4), suggesting that the rat had cleared *F. tularensis* either completely or to an undetectable level after 16 doses of levofloxacin. The exact cause of death in this animal is, therefore, unclear.

## 5. Conclusions

Together, survival, body weight, and bacterial burden data consistently demonstrated inhalational exposure to *F. tularensis* leads to severe tularemia and death in the Fischer 344 rat model of infection and that 1000 CFUs/animal is the optimal target exposure dose for testing potential therapeutics in the model. Furthermore, therapy with ceftobiprole medocaril is an effective treatment when administered by IV infusions at the reported dosing regimen mimicking the human exposure of the clinical dosing regimen. While the outcomes of this study are promising, further evaluation of ceftobiprole medocaril in a larger animal model of tularemia with a humanized dosing schematic will be a critical next step. Eventually, demonstrating efficacy in multiple animal models will be necessary for approval as an indication against pneumonic tularemia by the FDA Animal Rule [27]. Additionally, as this study used a Schu S4 submaster cell bank, the results may not represent all *F. tularensis* strains and could require replication to show biodiverse applicability. Herein, we have demonstrated sufficient justification for advanced testing and expect that successful development of ceftobiprole medocaril will enhance the diversity of therapeutics available in the US strategic national stockpile that could be used in response to an intentional release of aerosolized *F. tularensis*.

## Figures and Tables

**Table 1:** Inhaled exposure doses administered in Phase I (model refinement).

Phase I Group	Number of Animals	Target Exposure Dose (CFUs/Animal)	Actual Exposure Dose (CFUs/Animal)	Group Mean Body Weight (g)
1	10	100	377	167.90
2	10	1000	3515	167.72

**Table 2:** Minimum inhibitory concentration of ceftobiprole and ciprofloxacin against *F. tularensis* Schu S4 and *S. aureus* ATCC 29213.

Organism	Sample	Starting Inoculum Density (CFUs/mL)	Ceftobiprole MIC (µg/mL)	Ciprofloxacin MIC (µg/mL)
*F. tularensis* Schu S4	CDA	2.06 × 10^6^	<0.03	0.03
*F. tularensis* Schu S4	CDB	1.53 × 10^6^	<0.03	0.016
*S. aureus* ATCC 29213	CDA (QC organism)	4.90 × 10^6^	0.5	1
*S. aureus* ATCC 29213	CDB (QC organism)	1.38 × 10^7^	0.25	1
CDA: Challenge Day A; CDB: Challenge Day B; QC: Quality Control				

## Data Availability

Not applicable.

## Supplementary Materials

The following supporting information can be downloaded at: https://www.mdpi.com/article/10.3390/antibiotics12081337/s1, Table S1: Descriptive statistics for body weight by challenge group and day; Table S2: Descriptive statistics for body weight by treatment group and day; Table S3: Summary of statistical test results for mean shift from baseline for daily or weekly body weights in Phase II.
