# Supplementary material for: Ceftobiprole Medocaril Is an Effective Post-Exposure Treatment in the Fischer 344 Rat Model of Pneumonic Tularemia
Source: Antibiotics (Basel). 2023 Aug 19;12(8):1337. doi: 10.3390/antibiotics12081337 (PMC10451734; doi:10.3390/antibiotics12081337)
Supplement: Supplementary file 1 [file antibiotics-12-01337-s001.zip › antibiotics-2505727-supplementary.pdf]

Table S1. Descriptive statistics for body weight by challenge group and day.

Phase I Group	Day post-challenge	N	Arithmetic Mean (95% Confidence Interval) (g)
1	0	10	167.90 (164.22, 171.58)
	7	7	139.47 (134.58, 144.36)
	14	1	*135.80 (--)
2	0	10	167.72 (162.12, 173.32)
	7	4	134.13 (126.62, 141.63)

* N = 1; therefore, not a group mean at this time point

The actual weight range of all rats was 153.4 – 177.2 g.

Table S2. Descriptive statistics for body weight by treatment group and day.

Phase II Group	Day post-challenge	N	Arithmetic Mean (95% Confidence Interval) (g)
1	0	12	176.18 (172.72, 179.64)
	1	12	175.02 (171.11, 178.93)
	2	12	176.75 (173.15, 180.35)
	3	12	169.68 (165.32, 174.03)
	4	12	163.57 (159.63, 167.52)
	5	12	165.53 (161.77, 169.30)
	6	12	165.51 (161.65, 169.37)
	7	12	165.22 (161.03, 169.40)
	8	12	163.65 (158.78, 168.51)
	9	12	164.45 (159.29, 169.61)
	10	12	163.40 (159.19, 167.61)
	11	11	163.48 (159.85, 167.10)
	12	11	166.35 (161.82, 170.87)
	13	11	165.31 (161.38, 169.25)
	14	11	168.57 (165.08, 172.07)
	15	11	171.35 (167.28, 175.43)
	16	11	172.62 (168.32, 176.92)
	17	11	173.68 (169.35, 178.01)
	24	11	179.45 (173.26, 185.65)
	31	11	186.49 (181.04, 191.94)
2	0	12	175.91 (171.76, 180.06)
	1	12	176.41 (171.92, 180.90)
	2	12	176.89 (173.19, 180.60)
	3	12	170.08 (165.53, 174.63)
	4	12	167.46 (163.31, 171.62)
	5	12	170.60 (166.94, 174.26)
	6	12	170.27 (166.43, 174.12)
	7	12	170.89 (166.98, 174.81)
	8	12	170.21 (166.25, 174.17)
	9	12	170.70 (167.26, 174.15)
	10	12	170.95 (167.63, 174.27)
	11	11	170.74 (167.29, 174.20)
	12	11	174.85 (171.32, 178.37)
	13	11	174.45 (170.78, 178.11)
	14	11	174.79 (171.20, 178.38)
	15	11	176.10 (171.74, 180.46)
	16	11	175.94 (171.34, 180.53)
	17	11	176.44 (172.63, 180.25)
	24	11	182.88 (177.81, 187.95)
	31	11	184.23 (179.86, 188.60)
3	0	8	173.20 (168.56, 177.84)
	1	8	173.55 (169.48, 177.62)
	2	8	173.83 (169.77, 177.88)
	3	8	166.35 (161.80, 170.90)
	4	8	158.10 (154.61, 161.59)
	5	8	156.18 (152.12, 160.23)
	6	8	148.39 (143.51, 153.28)
	7	8	145.76 (140.81, 150.71)
	8	7	141.19 (136.44, 145.95)
	9	7	137.86 (133.61, 142.12)
	10	4	131.93 (121.57, 142.28)

Table S3. Summary of statistical test results for mean shift from baseline for daily or weekly body weights in Phase II.

Day post-challenge	Mean Shift from Baseline (g)<br>1	 2	 3	Group Effect P-Value	Group Comparison Estimate (Relationship) P-Value#
1	-1.16	0.50	0.35	0.1923	NS
2	0.57	0.98	0.63	0.8452	NS
3	-6.51 ↓	-5.83 ↓	-6.85 ↓	0.7984	NS
4	-12.61 ↓	-8.45 ↓	-15.10 ↓	0.0010*	4.16 (1<2) 0.0217<br>6.66 (3<2) 0.0010
5	-10.65 ↓	-5.31 ↓	-17.02 ↓	<0.0001*	5.34 (1<2) 0.0052<br>6.38 (3<1) 0.0028<br>11.71 (3<2) <0.0001
6	-10.67 ↓	-5.64 ↓	-24.81 ↓	<0.0001*	5.04 (1<2) 0.0078<br>14.14 (3<1) <0.0001<br>19.17 (3<2) <0.0001
7	-10.96 ↓	-5.02 ↓	-27.44 ↓	<0.0001*	5.95 (1<2) 0.0139<br>16.47 (3<1) <0.0001<br>22.42 (3<2) <0.0001
8	-12.54 ↓	-5.70 ↓	-31.29 ↓	<0.0001*	6.84 (1<2) 0.0036<br>18.76 (3<1) <0.0001<br>25.59 (3<2) <0.0001
9	-11.73 ↓	-5.21 ↓	-34.62 ↓	<0.0001*	6.53 (1<2) 0.0111<br>22.89 (3<1) <0.0001<br>29.42 (3<2) <0.0001
10	-12.78 ↓	-4.96 ↓	-39.70 ↓	<0.0001*	7.82 (1<2) 0.0003<br>26.92 (3<1) <0.0001<br>34.74 (3<2) <0.0001
11	-12.20 ↓	-5.93 ↓	-	0.0005*	6.27 (1<2) 0.0005
12	-9.33 ↓	-1.83	-	0.0004*	7.51 (1<2) 0.0004
13	-10.37 ↓	-2.22 ↓	-	<0.0001*	8.14 (1<2) <0.0001
14	-7.11 ↓	-1.88	-	0.0008*	5.22 (1<2) 0.0008
15	-4.32 ↓	-0.57	-	0.0277*	3.75 (1<2) 0.0277
16	-3.06 ↓	-0.74	-	0.1194	NS
17	-2.00 ↓	-0.24	-	0.1895	NS
24	3.78	6.21 ↑	-	0.4689	NS
31	10.81 ↑	7.56 ↑	-	0.0449*	3.26 (2<1) 0.0449

↑ Indicates the mean at the day was significantly greater than that at baseline (at the 0.05 level).

↓ Indicates the mean at the day was significantly less than at baseline (at the 0.05 level).

* Group effect was statistically significant at the 0.05 level.

# Cells contain all pairwise comparisons that were significant at the 0.05 level. The format within each cell is: (1) the mean difference, (2) the relationship between the corresponding pair of group means shown in parentheses, and (3) the Tukey-adjusted p-value. Note that all animals in the placebo group died on or before 10 days post-challenge, so comparisons to the treatment groups were not possible after 10 days post-challenge.

NS There were no significant pairwise differences at the 0.05 level.
